# Site and Strain-Specific Variation in Gut Microbiota Profiles and Metabolism in Experimental Mice

**DOI:** 10.1371/journal.pone.0008584

**Published:** 2010-01-05

**Authors:** Melissa K. Friswell, Helen Gika, Ian J. Stratford, Georgios Theodoridis, Brian Telfer, Ian D. Wilson, Andrew J. McBain

**Affiliations:** 1 Microbiology Research Group, School of Pharmacy and Pharmaceutical Sciences, The University of Manchester, Manchester, United Kingdom; 2 Experimental Oncology Research Group, School of Pharmacy and Pharmaceutical Sciences, The University of Manchester, Manchester, United Kingdom; 3 AstraZeneca, Department of Clinical Pharmacology, Drug Metabolism and Pharmacokinetics, Macclesfield, United Kingdom; 4 Department of Chemistry, Aristotle University of Thessaloniki, Thessaloniki, Greece; University of Hyderabad, India

## Abstract

**Background:**

The gastrointestinal tract microbiota (GTM) of mammals is a complex microbial consortium, the composition and activities of which influences mucosal development, immunity, nutrition and drug metabolism. It remains unclear whether the composition of the dominant GTM is conserved within animals of the same strain and whether stable GTMs are selected for by host-specific factors or dictated by environmental variables.

**Methodology/Principal Findings:**

The GTM composition of six highly inbred, genetically distinct strains of mouse (C3H, C57, GFEC, CD1, CBA nu/nu and SCID) was profiled using eubacterial –specific PCR-DGGE and quantitative PCR of feces. Animals exhibited strain-specific fecal eubacterial profiles that were highly stable (*c.* >95% concordance over 26 months for C57). Analyses of mice that had been relocated before and after maturity indicated marked, reproducible changes in fecal consortia and that occurred only in young animals. Implantation of a female BDF1 mouse with genetically distinct (C57 and Agoutie) embryos produced highly similar GTM profiles (*c.* 95% concordance) between mother and offspring, regardless of offspring strain, which was also reflected in urinary metabolite profiles. Marked institution-specific GTM profiles were apparent in C3H mice raised in two different research institutions.

**Conclusion/Significance:**

Strain-specific data were suggestive of genetic determination of the composition and activities of intestinal symbiotic consortia. However, relocation studies and uterine implantation demonstrated the dominance of environmental influences on the GTM. This was manifested in large variations between isogenic adult mice reared in different research institutions.

## Introduction

The gastrointestinal tract microbiota (GTM) of mammals supports a microbial ecosystem, which at up to 10^14^ cells, outnumbers somatic cells by at least one order of magnitude. The interplay between GTM and host is complex, with diverse positive and potentially negative implications for host health in the short and longer-term. Intestinal colonization-resistance for example, may reduce the incidence and severity of intestinal infections through limitation of adhesion sites and growth substrates and by the accumulation of inhibitory metabolites [1]. It is also clear that the GTM plays an important role in the development of the immune system from birth onwards [2] and in gastrointestinal mucosal development [3], [4], [5], [6]. The composition and metabolism of the GTM affects other fundamental host processes including drug metabolism, where the absorption and bioavailability of drugs and their metabolites may be altered through prokaryotic biotransformation [7], [8]. Furthermore, host nutritional status may be markedly influenced by the composition and activities of the GTM since it is apparent that the ratio of Bacteroidetes to Firmicutes is decreased in obese individuals and genetically obese mice harbour an “obese microbiome”, with a transferable elevated capacity for energy sequestration [9].

Several recent investigations have demonstrated considerable integration of prokaryotic and mammalian metabolism with respect to polysaccharide metabolism [10], [11], [12] and other host processes [13], [14] which is strong evidence for co-evolution of host and microbiota. The GTM can therefore be viewed as a versatile prokaryotic metabolic organ [10], [11], [15].

Evidence is emerging to suggest a considerable degree of individuality and temporal stability within human and murine GTMs. Various studies using PCR-DGGE and cluster analyses of human [16], [17] and murine GTMs [18], [19] for example, have indicated the occurrence of host-specific predominant mucosa-associated and luminal bacterial community profiles.

Factors responsible for the development, maintenance and stability of individual-specific GTMs remain unclear. It is possible that, through the inherent stability and colonisation resistance of complex microbial climax communities, GTMs may maintain specific profiles through microbial competition, particularly during colonization [20]. However, since the microbiota exists in close proximity with mammalian tissues, there are many host factors host that may also influence the diversity, richness and stability of the microbiota. Mammalian tissues can sense and coordinate appropriate immunological responses to symbiotic and pathogenic bacteria using Toll-like and Nod-like receptors (TLRs and NLRs) [21], [22]. The involvement of TLRs and NLRs in gut homeostasis has been additionally suggested by the presence of polymorphisms in TLR and NOD2 genes in patients with inflammatory bowel disease [23]. Other variables known to broadly influence the composition of the GTM include age, where distinct microbiota profiles have been associated with the young and aged individuals [24]; diet which has a measurable but apparently limited influence [20], [25], the use of antibiotics [26] and other drugs [27] and “lifestyle” [28].

Despite a considerable increase in interest in the function and natural history of the GTM and in strategies for its therapeutic compositional manipulation, insight into the broad contributions of host genetics *versus* environmental factors upon development, composition and maintenance of the GTM remains incomplete and there are relatively few reports addressing this in the literature, probably due to difficulties associated with experimentally separating environmental from genetic influences.

The possible involvement of host genotype, particularly as it relates to immuno-phenotype, has been frequently postulated as a major influence on GTM composition and stability although this has been difficult to prove. For example, recent studies have investigated the possible correlation between host genetic relatedness and fecal microbial profiles in human adult twins. Zoetendal *et al.* [6] used PCR-DGGE-based fecal profiling to demonstrate a correlation between overall genetic relatedness and similarity indices of abundant fecal microbiota 16S rRNA sequence variants. Similarly, Stewart *et al.* [29] profiled fecal bacterial populations and calculated of the degree of similarity in the predominant fecal microbiota of identical twin pairs, fraternal twin pairs and unrelated, paired controls. Since the highest levels of similarity was found in genetically identical twins, with significant differences evident between the identical and fraternal twins, the authors concluded host genetics influenced the composition of the dominant eubacterial population in children. Dicksved et al. [30] investigated the GTM profiles in 10 monozygotic twin pairs where one or both had Crohn's disease and eight healthy twin pairs and showed that GTMs were more similar between healthy twins than between twins with CD, especially when these were discordant for the disease. The authors concluded that “genetics and/or environmental exposure during childhood, in part, determine the gut microbial composition”.

Despite the obvious usefulness of studies using human twins, they do not present a definitive means of separating genetic from environmental influences due to the frequent confounding influence of close social and familial contact. In the Stewart study for example [29], the genetically-related volunteers were living in the same home environment at the time of sample collection.

Further evidence for the importance of host genetics in the determination of host-microbe interaction is provided by a recent study by Khachatryan *et al.* [31] who profiled the GTMs of subjects with the auto-inflammatory disorder, familial Mediterranean fever and showed that significant changes in GTMs occurred during inflammatory episodes according to sequence analyses and FISH. Importantly however, the allele-carrier status of individuals for the genes associated with this disease was a significant determinant of GTM composition, even in remission.

Whilst the environment represents a continuous microbial challenge to the GTM, it is believed that external influences upon the microbial composition of the GTM are greatest in nascent microbiotas, following birth. In new-borne mammals, the sterile intestine represents a readily colonisable environment, being subject to microbial immigration from sources such as the birth canal and faecal material [32]. Bacteria associated with breast milk have been shown to represent a source of bifidobacteria for infant GTMs [33] and an extensive metagnomic study of human infants has indicated compositional and temporal patterns of the microbial communities which vary widely between individuals, leading to the development of a characteristic adult GTM profile after *c.* 12 months [34]. In the same study, temporal patterns of consortial development between twins were “strikingly parallel”; an observation used to support the hypothesis that “incidental environmental exposures” are responsible for individualised GTMs.

Other environmental variables may significantly influence the composition of the GTM, including antibiotic use where it has for example been shown that oral ciprofloxacin dosing alters the abundance of approximately one third of GTM taxa, decreasing the richness and diversity with profiles returning to re-treatment states for most taxa 4 weeks after the end of treatment [35].

In the current investigation, we have utilized six strains of highly inbred experimental mice, which, through considerable genetic relatedness between siblings and amenability to environmental manipulation, represent useful models for the evaluation of intrinsic and extrinsic influence upon the GTM composition. In order to study the influence of host genetics *vs.* environmental influences; i) the GTMs of age- and gender-matched mice were profiled, including isogenic murine strains maintained within separate locations and within two different research establishments; ii) relocation studies were conducted whereby immuno-competent and severely immuno-compromised mice were moved in order to assess the contributions of immunophenotype and consortial-intrinsic colonization resistance and finally, iii) the implantation of a female BDF1 mouse with embryos from two genetically distinct mouse strains effectively differentiated between the effects of genetic and environmental influences upon fecal eubacterial profiles and urinary metabolites in co-gestated animals. Better understanding of the factors that influence the stability and composition of the murine GTM is likely to have significant implications for use of experimental animal models but may also be of fundamental importance in terms of general understating of the development and composition of mammalian (and thus human) GTMs.

## Results and Discussion

The composition and activities of the mammalian GTM significantly influences host physiology including immune function [36], adiposity [9], [37] and drug metabolism [7]. Whilst these processes fundamentally affect the animal and human health, they may also represent important and largely overlooked variables when designing animal experiments and interpreting associated data. In the current investigation, the hypothesis that murine GTM composition and metabolism is dictated principally by genetic factors was tested using various highly inbred mouse strains and a reproducible DNA fingerprinting technique (PCR-DGGE), combined with quantitative PCR and urinary metabolite analyses for compositional and metabolic analyses, respectively. Uniquely, embryos of two distinct strains of mice (C57 and Agoutie) were surgically implanted into a surrogate BDF1 strain mother, thus enabling GTM development to be investigated within different genetically distinct strains of mice under essentially identical environments.

Better understanding of strain and location-dependant variation in murine GTMs has obvious implications for the use of animals as experimental models. Additionally, however fecal material from the highly inbred mouse strains can be used to represent paradigm vertebrate microbiotas which could provide fundamental insights into factors influencing the development and maintenance of GTMs.

### Inter-strain variation in GTM profiles

**Figure 1:**
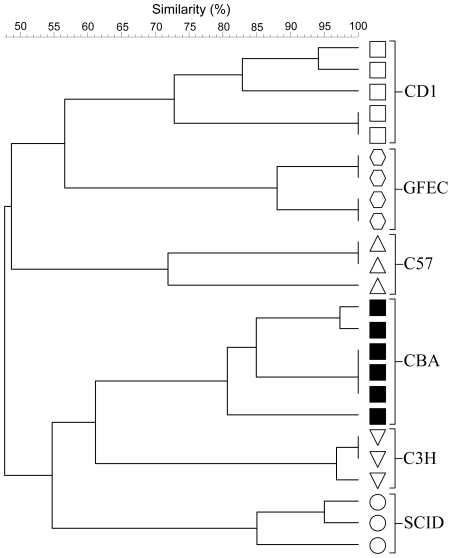
Strain-dependent clustering of mouse GTMs. UPGMA cluster analysis of fecal DGGE profiles from six genetically distinct strains of mice. Acronyms refer to mouse strain. All samples originated from distinct mice.

**Figure 2:**
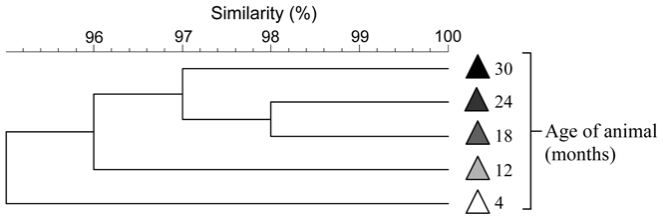
Temporal GTM stability within a single C57 strain mouse sampled over a 30-month period. (UPGMA cluster analysis of fecal DGGE profiles).

Cluster analysis of fecal denaturing gradient gel electrophoresis (DGGE)-derived microbial fingerprints was used to compare the GTMs of mature female mice from the following mouse strains: C3H, C57, GFEC, CD1, CBA nu/nu, and SCID, which were selected as paradigm genetically distinct mice. The use of CBA nu/nu and SCID mice additionally enabled specific immune-impairment to be studied since these animals lack T cells and T and B cells, respectively. GTM fingerprints from replicate (n = 3–6) animals, housed at the University of Manchester (designated environment A) clustered together, based on mouse strain with inter-strain concordance, ranging from 71 to 100% and intra-strain concordance between 50 and 55% (Fig. 1). This observation was largely corroborated by quantitative PCR (Table 1) where inter-strain variation was apparent, particularly for total fusobacteria, bifidobacteria and lactobacilli. Importantly, quantitative PCR analysis showed variation in numbers of organisms present in individual mice of the same strain were not significant for 37/49 values (Table 1). Animals were also shown to retain a highly stable, mature GTM over time (Fig. 2). Whilst these data were suggestive of genetic determination of GTM profiles, further analyses was necessary to differentiate between environmental and genetic factors, since individual mouse strains were housed together with like strains and familial GTM profiles could be perpetuated through maternal contact and by coprophagia.

**Table 1:** 16S rRNA copy numbers of selected classes of intestinal bacteria in the feces of different strains of mice.

Mouse strain	Total bacteroidetes	Total enterobacteriaceae	*Clostridium perfringens*	Total bifidobacteria	Total lactobacilli	Total fusobacteria	Total enterococci
GFEC	10.17 (1.14)^b^	6.24 (2.41)^c,f^	7.30 (0.48)^d,f,g^	1.74 (1.64)^b,c,g^	*8.63 (2.37)^c,d,e,f^	*7.93 (6.89)^c,d,f^	*3.39 (1.13)^f^
C57	11.93 (1.15)^a,d,e,f^	*5.61 (2.35)^f^	7.47 (0.05)^d,f,g^	*6.52 (3.15)^a,c,d,e,f^	10.08 (1.11)^d,e,f^	3.10 (0.94)^c,d,e^	*3.82 (1.33)^f^
CD1	10.43 (0.48)^d,e,f^	4.51 (2.08)^a,g^	6.91 (0.88)^d,f^	0.87 (0.59)^a,b,d,e,f^	10.67 (1.90)^a,d,e,f^	1.73 (0.57)^a,b,f,g^	3.32 (0.70)^f^
CBA	9.56 (0.12)^b,c,g^	4.05 (0.25)	5.54 (0.99)^a,b,c,e^	1.38 (0.58)^b,c,g^	5.00 (1.17)^a,b,c,g^	0.91 (0.58)^a,b,e,g^	*2.67 (1.33)^g^
SCID	*8.60 (2.05)^b,c,g^	5.89 (2.75)^f^	6.94 (0.67)^d,f^	1.50 (1.18)^b,c,g^	5.03 (0.59)^a,b,c,g^	3.39 (1.98)^c,d,f^	2.86 (0.27)^f^
C3H (Manchester)	9.42 (0.51)^b,c,g^	3.45 (0.54)^a,b,e,g^	5.08 (0.28)^a,b,c,e,g^	*2.81 (1.37)^b,c^	4.78 (1.00)^a,b,c,g^	*0.45 (1.12)^a,b,e,g^	2.27 (0.28)^a,b,c,e,g^
C3H (Stanford)	10.86 (0.81)^d,e,f^	*5.91 (1.92)^c,f^	6.28 (1.33)^a,b,f^	4.49 (1.51)^a,c,d,e^	8.96 (0.83)^d,e,f^	*4.60 (4.54)^c,d,f^	3.63 (0.66)^d,f^

Data are mean values of log_10_ copy numbers/g feces (animals, n = 3; technical replicates n = 3). Numbers in parenthesis are standard deviation values. Superscript letters denote statistically significant differences (p<0.01) between mouse strains; a: GFEC; b: C57; c: CD1; d: CBA; e: SCID f: Manchester C3H; and g: Stanford C3H. *Denotes significant differences within replicate animals of the same strain.

### Effect of relocation on murine GTMs

**Figure 3:**
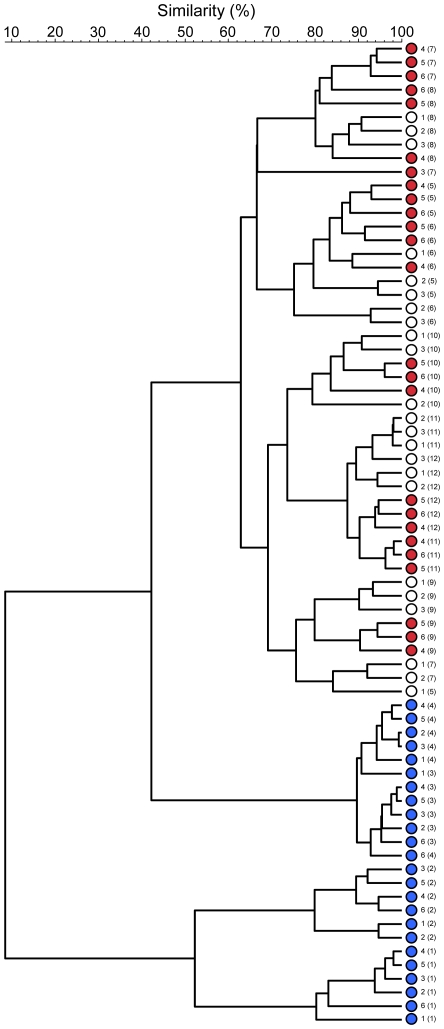
Effect of relocation on the GTM profiles of highly immunocompromised SCID mice (UPGMA cluster analysis of fecal DGGE profiles). All animals were housed in environment A until 8 weeks of age. Samples obtained during this period are designated by blue symbols. Red symbols represent fecal samples obtained from animals that remained in the original environment (A) (sampled in the animals over 8 weeks of age); open symbols represent animals relocated to environment B. Numbers indentify individual animals; numbers in parenthesis give the age of animal in weeks.

**Figure 4:**
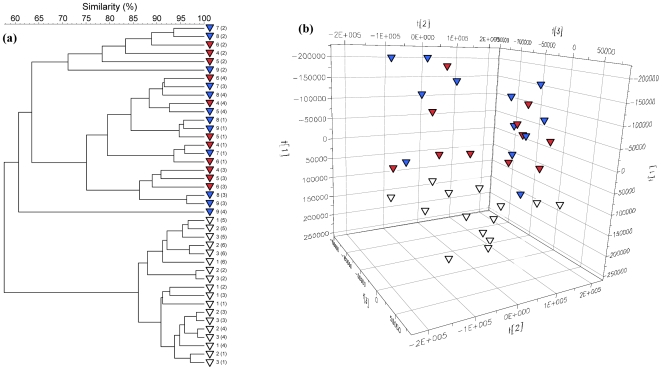
Age-dependent effects of relocation on the GTM profiles and urinary metabolite profiles of C3H mice. (a) UPGMA cluster analysis of fecal DGGE profiles. Blue symbols represent fecal samples obtained from animals housed in the original environment (A). Samples obtained from mice relocated at 4 weeks of age are represented by open symbols; red symbols represent animals relocated to environment B at over 8 weeks of age. Numbers identify individual animals; numbers in parenthesis give the age of animal in weeks and (b) UPLC-MS PCA results from the analysis of urine samples from these animals showing the effect of relocation on metabolite profiles.

The immune system of rodents is considered immature at four weeks of age, reaching maturity by approximately 8 weeks [38]. In order to investigate the stability of GTMs, the effect of environmental change was studied in highly immunocompromised SCID mice; selected from a line that had been bred and maintained in the same unit within the University of Manchester (environment A) for 6 years. These animals were relocated to an alternative room (environment B) within the same institution at 8 weeks of age (n = 3). Age-matched animals were also maintained in their original environment. All animals underwent changes in GTM profiles between 4 and 8 weeks of age but at maturity, the GTMs of relocated animals did not markedly differ from those that had not been moved (Fig 3). This experiment was also repeated for C3H mice with the elaboration that animals were relocated at either *c.* 4 or 8 weeks of age. This resulted in marked changes in GTM profiles that were only manifested in immature animals, as evidenced by the distinct clustering of GTM profiles in these animals (Fig. 4a). Additionally, urinary metabolite profiles for C3H mice moved at 8 weeks of age and those that remained in the original environment clustered together and were distinct from those moved at four weeks of age, thus corroborating the DGGE data (Fig 4b). Examination of the UPLC-MS data revealed a complex pattern of ions/metabolites increasing or decreasing in intensity in the group relocated at four weeks of age compared to those in the other two groups indicating a difference in metabolite patterns between the two groups. Confirmation of the characteristic GTM profiles associated with each animal, together with high degrees of microbiota stability of mature animals suggested a possible role for host-specific factors but could also be attributed to colonization-resistance of mature GTMs, resulting in the age-dependant variations in the influence of environmental change. It was therefore, necessary to more effectively differentiate between environment and genetic factors in order to better differentiate between these variables.

### GTM development in genetically distinct, uterine-implanted mice

**Figure 5:**
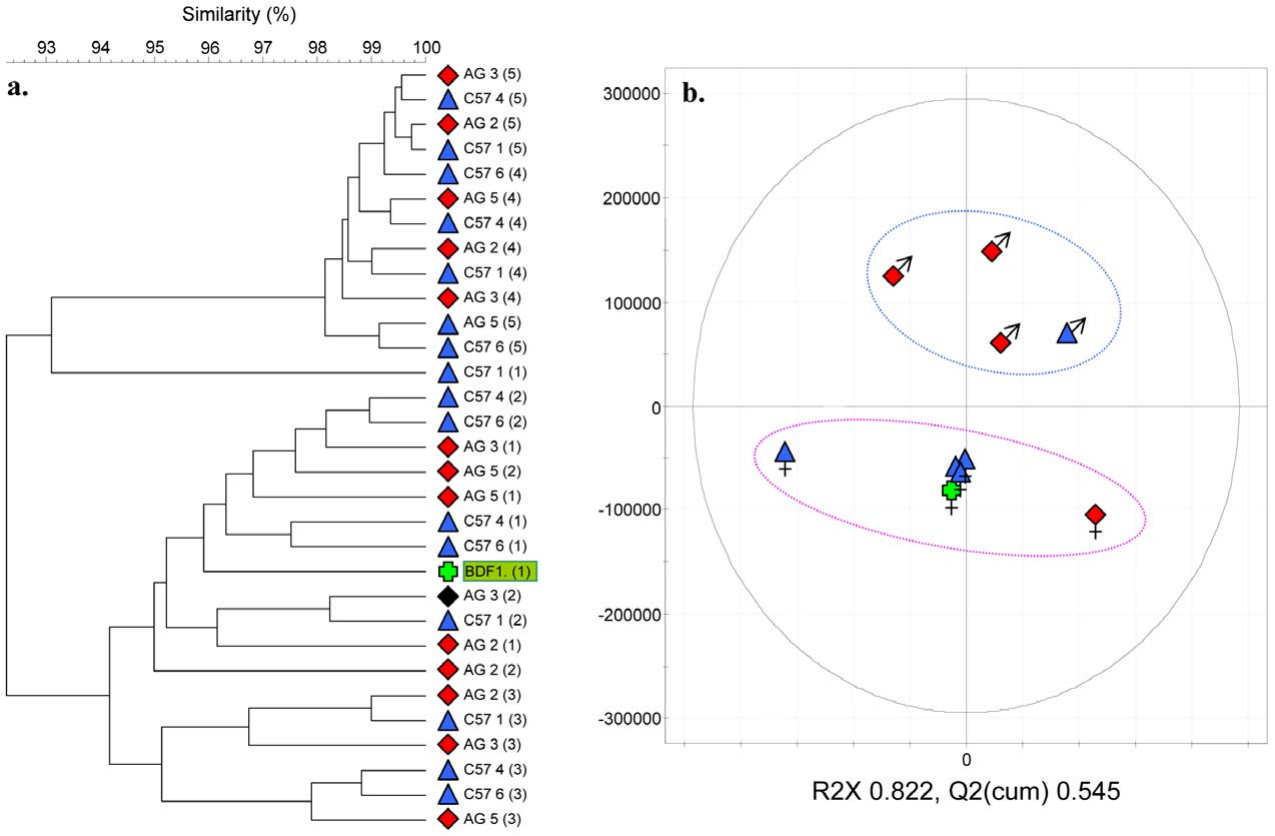
GTM development in genetically distinct, uterine-implanted mice. (a) UPGMA cluster analysis of fecal DGGE profiles; (b) PCA score plot of the two first PCs of analysis of UPLC-MS urine analysis data set (gender is indicated). The model describes 82% of the variation. Urinary metabolite profiles cluster on the basis of gender (blue cluster boundary, males; pink cluster boundary, females) but not genotype. C57 (blue triangles) and Agoutie; AG (red diamonds) gestated in a BDF1 strain mother (green cross).

In order to broadly determine the relative contribution of the environment on GTM microbial diversity, an experiment was carried out whereby a female BDF1 was implanted with three Agouti (Ag) and three C57 embryos. Fecal samples were collected from the offspring and mother at weekly intervals following weaning (3 weeks of age) until 10 weeks of age. Fostered, genetically distinct progeny (Ag and C57) could not be differentiated from the BDF1 mother based on GTM fingerprints (Fig. 5a), with dendrogram concordance of in excess of 93%. Furthermore, metabonomic analysis of urine samples from these animals indicated that whilst animals could be differentiated based on their gender, mouse genotype was not an effective differentiator (Fig. 5b). Concordance within GTM bacterial profiles was therefore also manifested metabolically. These observations suggest that environment is a dominant influence upon the GTM profiles since progeny would otherwise have maintained strain-specific GTM profiles.

### Murine GTMs of isogenic mice from distinct research centers

**Figure 6:**
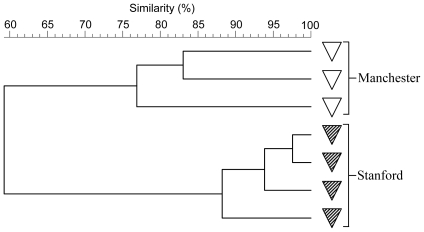
Institution-specific GTMs in C3H mice. A UPGMA dendrogram generated from fecal bacterial fingerprints of C3H mice housed at within UK (University of Manchester) and USA (Stanford University) institutes. All sample originated from distinct mice.

In order to examine the practical implications of the purported major influence of environment upon GTM profiles and for corroboration, feces from C3H mice obtained from Stanford University and The University of Manchester breeding houses was subjected to PCR-DGGE and cluster analysis. Data generated in this manner indicated that mice from the Manchester breeding house had substantially different GTM profiles from C3H mice at Stanford University (c. 60% concordance for Manchester vs. Stanford compared to >80% inter-strain concordance; Figure 6) despite being nominally identical strains. The Manchester and Stanford C3H breeding colonies originated from the same source, having been gifted by the National Institutes of Health in 1947 but over time, unique GTMs appear to have developed in the individual colonies, probably because of because consortial drift and distinct local environmental conditions.

### Conclusions

The major role of the GTM in the metabolism of dietary residues and xenobiotics [7] and its associated influence of host physiology and metabolism [9], [14] means that marked variation within GTMs between research institutions, which is apparently more strongly influenced by the environment than by host genotype, has implications for many areas of research. Animal models are currently used to assess the pharmacokinetics, metabolism and toxicology of new drugs etc. Differences in GTMs associated with the maintenance of animals within distinct research centers could have obvious, but to date, poorly understood effects on experimental outcomes. Such variation may for example, significantly influence drug metabolism within apparently identical mouse strains [5]. Specific metabonomic studies have shown that individual, pre-dose urinary metabolic phenotypes (which are strongly influenced by the microbiota [39] can be used to predict the metabolic fate and toxicity of drugs and other xenobiotics in animals [8] and the urinary metabolic phenotype of human volunteers was recently found to predict the metabolic fate of acetaminophen (paracetamol) based on the quantity of urinary p-cresol, a product of gut microbial metabolism [40], [41].

Whilst other reports have suggested that host genetics are primarily responsible for the composition of the GTM in humans and animals [6], [29] some of these have utilized fraternal and identical human twins and sibling groups. Whilst such approaches are laudable, differentiation between environmental and genetic factors remains a problem. Other investigations have however, indicated a complex interplay between genetics and environment. For example, a study involving reciprocal transplantations of GTMs between germ-free zebra fish and mice has indicated that host-factors are involved in the selection of GTM profiles which, although resembling those of the donor animal with respect to bacterial lineages, assumed relative abundances more closely resembling the normal GTM profile of the recipient host [42]. Another key investigation used a comprehensive, network-based analysis of fecal 16S rRNA genes sequences from humans but also from 59 other mammalian species to elucidate major influences such as diet and host phylogeny upon the GTM composition and to gain insight into the likely evolution of such associations [43]. GTM communities were more similar between conspecific hosts than between different species and when conspecific hosts lived separately, significant clustering was still apparent in some (for example, between two baboons living on different continents; one in the wild, one in captivity), but not all cases. Highly significant clustering was also apparent according to diet which could be analysed as a variable independently of host phylogeny. Interpersonal differences between humans appeared to be lower than differences between distinct mammalian species.

In the current study, the use of animal models has suggested that the environment is a major factor in the determination of the GTM profiles of mice. This is evidenced by the apparent strain-dependence of GTM compositional and metabolic profiles, which were absent when genetically distinct mice were gestated and reared by the same mother and by GTM variation within isogenic mice derived from different research institutions. From a practical perspective, differences in GTM profiles and metabolism within putatively isogenic mice could result in significant unforeseen variation in the effects of drug treatment in experimental animals and ultimately in humans.

## Materials and Methods

### Ethics

All procedures used in this study incorporated the 1998 United Kingdom Coordinating Committee on Cancer Research Guidelines and were in compliance with The Scientific Procedures Act of 1986. Studies were approved by the Home Office Inspectorate and the University of Manchester Ethics Care Committee under PPL 40/2328. Animals were maintained using the highest possible standard of care and priority was given to their welfare above experimental demands at all times.

### Mouse strains

All mice used within this study were housed within the University of Manchester animal facilities. Mouse strains used in the work were: C3H, C57BL6, Agouti (C57BL6×BALB/C), GFEC (A transgenic mouse on a C57BL6 background, engineered to express GFP tagged glial fibrillary acidic protein), BDF1 (C57BL6×DBA2, [B-cell deficient] used for super ovulation and embryo implantation), CBA nu/nu and CD-1 nu/nu (both T-cell deficient), and SCID mice (T- and B-cell deficient). All strains are bred in-house with the exception of the C57, CD-1 nu/nu and SCID mice that were purchased at >4 weeks old from Charles River, UK.

### Murine relocation studies

Mature Mice (n = 6) from two distinct strains (C3H and CBA – in total twelve mice) were selected for this experiment. The mice were divided into two groups (n = 3 in each group) and identified by means of ear tagging. One group of mice were relocated to environment B (quarantine area of another animal house) upon reaching maturity (8 weeks of age), whilst the other group remained in the original breeding environment (environment A). Fecal samples were collected weekly from all individuals and subjected to 16S PCR-DGGE. Fingerprints were sorted using cluster analysis. Mice were transported and housed in individual autoclaved housing units within the quarantine area of environment B, and fed the same autoclaved food. This was repeated from immature animals whereby mice (n = 3) of two genetically distinct strains (C3H and CBA – total 6 mice) were selected for the experiment. Upon weaning (4 weeks of age), all mice were moved to environment B. In all cases, fecal samples and urine were collected weekly from all individuals and archived at −70 for subsequent analysis.

### Implantation of genetically distinct mouse embryos

Agoutie and C57 mouse embryos at the blastocyte stage were implanted into a pseudo-pregnant BDF1 strain mother. Mice were born naturally and allowed to remain with the surrogate mother until weaning. At this stage the mother was removed and sacrificed for health screening. One fecal sample was collected from the surrogate mother before she was removed and fecal samples were collected from the offspring from weaning until nine weeks of age.

### PCR-Denaturing Gradient Gel Electrophoresis (DGGE) of fecal samples

Fecal samples were collected from individual mice directly into a sterile Eppendorf tubes, weekly from 4 weeks of age. Samples were then frozen within 2 hours of receipt. DNA was extracted using a Qiagen stool mini DNA extraction kit (Qiagen, Sussex, UK) with the added step of using a mini bead beater to homogenize the sample. Glass beads were added to the feces with sterile buffer (provided in Qiagen kit) and mixed for 90s. The amount and quality of DNA extracted was estimated by electrophoresis of 5-µl aliquots on a 0.8% agarose gel and comparison to a molecular weight standard (stained with ethidium bromide). DNA extracts were stored at −60°C prior to analysis in nuclease-free containers.

The V2-V3 region of the 16S rRNA gene (corresponding to positions 339 to 539 of Escherichia coli) was amplified with eubacterium-specific primers HDA1-GC (5′-CGC CCG GGG CGC GCC CCG GGC GGG GCG GGG GCA CGG GGG GAC TCC TAC GGG AGG CAG CAG T-3′) and HDA2 (5′-GTATTA CCG CGG CTG CTG GCA C-3′) as previously described (40). The reactions were performed in 0.2-ml tubes with a DNA thermal cycler (model 480; Perkin-Elmer, Cambridge, United Kingdom). In all cases, reactions were carried out with Red Taq DNA polymerase ready mix (25 µl; Sigma, Poole, Dorset, United Kingdom), HDA primers (2 µl of each, 5 µM), nanopure water (16 µl), and extracted community DNA (5 µl, corresponding to ca.10 ng). The thermal program was 94°C (4 min) followed by 30 thermal cycles of 94°C (30 s), 56°C (30 s), and 68°C (60 s). The final cycle incorporated a 7-min chain elongation step (68°C). Electrophoresis was carried out at 150 V and 60°C for approximately 4.5 h. Gels were stained with SYBR Gold stain [diluted to 10^−4^ in 1× TAE; Molecular Probes (Europe), Leiden, The Netherlands] for 30 min. Gels were viewed under U.V illumination and RAW images captured using a Canon D60 DSLR camera with a 50mm macro lens with UV filter attached.

### Cluster analyses

Gel images were aligned using Adobe Photoshop 6.5 (Adobe, CA, USA) aided by running common samples on multiple gels, to allow comparison of more than one gel. Gel images were then analysed using Bionumerics software (Applied Maths, Sint-Martens Latem, Belgium). Lane boundaries were applied to gels and images which were optimised to reduce the background noise and smiling of lanes (where applicable). Automatically detected bands (checked manually) were used to create matching profiles for lanes (in relation to each other). The matching profiles for each lane were used to produce an Unweighted Pair Group Method with Arithmetic Mean (UPGMA) dendrogram. The UPGMA algorithm weighs each lane being analysed equally and computes the average similarity or dissimilarity of each lane to an extant cluster. The dendrogram that is produced using this method can then be used to observe clustering patterns between different lanes.

### Quantitative PCR of major groups within different strains of mice

Fecal samples from six strains of mice (n = 3 of each strain) were subject to Q-PCR using methods as described in Bartosch *et al* (2004) Primers sets, designed to specifically target lactobacilli, fusobacteria, enterobacteriaceae, enterococci, *Clostridium perfringens*, bifidobacteria and total bacteroidetes are given in Table 2. The copy number per ml of the target gene was calculated for each sample using standards of known copy numbers. Data were analysed statistically using a one way ANOVA test to look for significant differences in numbers of organisms between strains of mice. ANOVA testing was also used to determine any significant differences in numbers of organisms between individual mice of the same strain. Copy number values were calculated using Microsoft Excel. Data was statistically analysed using the Kruskal Wallace statistical significance test in the Statsdirect programme (Cheshire, UK). Mean values were logged and the standard deviation were also calculated using Statsdirect.

**Table 2:** Primers used for quantitative PCR.

Primer target	Amplicon size (bp)	Oligonucleotide sequence (5′ - 3′)	Annealing temp. (°C)	Reference
*Bacteroides–Prevotella– Porphyromonas*	140	F: GGTGTCGGCTTAAGTGCCATR: CGGA(C/T)GTAAGGGCCGTGC	68	Rinttila *et al.* [44]
*Bifidobacterium* spp.	243	F: TCGCGTC(C/T)GGTGTGAAAGR: CCACATCCAGC(A/G)TCCAC	58	Rinttila *et al* [44]
*Enterococcus* spp.	144	F: CCCTTATTGTTAGTTGCCATCATR: ACTCGTTGTACTTCCCATTGT	61	Rinttila *et al.* [44]
*Fusobacterium* spp.	273	F: CCCTTCAGTGCCGCAGTR: GTCGCAGGATGTCAAGAC	54	Rinttila *et al.* [44]
Enterobacteriaceae	195	F: CATGACGTTACCCGCAGAAGAAGR: CTCTACGAGACTCAAGCTTGC	63	Bartosch *et al.* [45]
*Clostridium perfringens*	105	F: CGCATAACGTTGAAAGATGGR: CCTTGGTAGGCCGTTACCC	55	Wise & Siragusa [46]
*Lactobacillus* group	341	F: CACCGCTACACATGGAGR: AGCAGTAGGGAATCTTCCA	58	Martínez *et al.* [47]

### Metabolic profiling

Urine samples, collected weekly and stored at −20°C were diluted with water (1∶3) and analysed by Ultra performance liquid chromatography (Waters, USA) linked to a Q-Trap 4000 mass spectrometer (Applied Biosystems, UK). Chromatography was performed on an Aqcuity 100×2.1 mm I.D. column (Waters, USA) at 50°C and a flow rate of 0.4 mL/min using reversed-phase elution with water and acetonitrile (both containing 0.1% formic acid v/v) over 10 min. Pooled samples were prepared as quality controls (QCs) and these were analysed between the samples. Mass scan data data (100–850 amu) were collected in +ve and −ve ESI mode. The raw data were extracted with MarkerView. Multivariate data analysis was performed with Simca P (Umetrics, Sweden; Principal Components Analysis (PCA) and Orthogonal Partial Least Squares Analysis (OPLS) were applied to project the data).
